# Retraction of: Oral Progenitor Cell Line-Derived Small Extracellular Vesicles as a Treatment for Preferential Wound Healing Outcome

**DOI:** 10.1093/stcltm/szag044

**Published:** 2026-05-18

This is a retraction of: Rob Knight, Emma Board-Davies, Helen Brown, Aled Clayton, Terence Davis, Ben Karatas, James Burston, Zsuzsanna Tabi, Juan M Falcon-Perez, Stephen Paisey, Phil Stephens, Oral Progenitor Cell Line-Derived Small Extracellular Vesicles as a Treatment for Preferential Wound Healing Outcome, *Stem Cells Translational Medicine*, Volume 11, Issue 8, August 2022, Pages 861–875, https://doi.org/10.1093/stcltm/szac037

In January 2025, the journal was alerted by the corresponding author to concerns regarding the animal protocol related to the in vivo studies that are closely interwoven within the in vitro data throughout the above-mentioned article. The journal subsequently investigated, and the corresponding author has confirmed there were institutional issues with the animal protocol. This resulted in a loss of confidence in the results and conclusions reported in the article. For these reasons, the corresponding author and Editors have decided to retract this article.

